# Electrical Transport Properties of PbS Quantum Dot/Graphene Heterostructures

**DOI:** 10.3390/nano14201656

**Published:** 2024-10-16

**Authors:** Haosong Ying, Binbin Wei, Qing Zang, Jiduo Dong, Hao Zhang, Hao Tian, Chunheng Liu, Yang Liu

**Affiliations:** 1 Department of Physics, Harbin Institute of Technology, Harbin 150001, China; 24b911011@stu.hit.edu.cn (H.Y.); 19b911015@stu.hit.edu.cn (J.D.); 19b911016@stu.hit.edu.cn (H.Z.); tianhao@hit.edu.cn (H.T.); 2 Institute of System Engineering, Academy of Military Sciences, Beijing 100191, China; weibb.2009@tsinghua.org.cn (B.W.); liuchunheng@126.com (C.L.)

**Keywords:** PbS quantum dots, graphene, heterostructure, electrical transport

## Abstract

The integration of PbS quantum dots (QDs) with graphene represents a notable advancement in enhancing the optoelectronic properties of quantum-dot-based devices. This study investigated the electrical transport properties of PbS quantum dot (QD)/graphene heterostructures, leveraging the high carrier mobility of graphene. We fabricated QD/graphene/SiO_2_/Si heterostructures by synthesizing p-type monolayer graphene via chemical vapor deposition and spin-coating PbS QDs on the surface. Then, we used a low-temperature electrical transport measurement system to study the electrical transport properties of the heterostructure under different temperature, gate voltage, and light conditions and compared them with bare graphene samples. The results indicated that the QD/graphene samples exhibited higher resistance than graphene alone, with both resistances slightly increasing with temperature. The QD/graphene samples exhibited significant hole doping, with conductivity increasing from 0.0002 Ω^−1^ to 0.0007 Ω^−1^ under gate voltage modulation. As the temperature increased from 5 K to 300 K, hole mobility decreased from 1200 cm^2^V^−1^s^−1^ to 400 cm^2^V^−1^s^−1^ and electron mobility decreased from 800 cm^2^V^−1^s^−1^ to 200 cm^2^V^−1^s^−1^. Infrared illumination reduced resistance, thereby enhancing conductivity, with a resistance change of about 0.4%/mW at a gate voltage of 125 V, demonstrating the potential of these heterostructures for infrared photodetector applications. These findings offer significant insights into the charge transport mechanisms in low-dimensional materials, paving the way for high-performance optoelectronic devices.

## 1. Introduction

The integration of PbS quantum dots (QDs) with graphene represents a significant advancement in enhancing the optoelectronic properties of QD-based devices [1,2,3]. The combination of PbS QDs, known for their tunable bandgap and high light absorption [4,5], with graphene, which is recognized for its high carrier mobility and excellent electrical conductivity [6], offers substantial potential for improving performance in applications such as photodetectors [1], solar cells [7], and optoelectronic transistors [8]. To optimize the performance of PbS QD/graphene heterostructures and to design optoelectronic devices with high efficiency, it is crucial to understand their electrical transport properties, which are influenced by various factors, including temperature, light intensity, and wavelength. Understanding the influence of these factors is essential to achieve efficient charge transfer and transport within the heterostructure, thereby enhancing device performance. Furthermore, this knowledge provides insights into the fundamental mechanisms of charge transfer and transport in low-dimensional materials, which is valuable for the development of new optoelectronic materials and devices.

PbS has emerged as a leading material for infrared photodetectors due to its tunable peak-response wavelength, ranging from 600 nm to 3000 nm, enabling the detection of light in the near-infrared and mid-infrared [9]. PbS QDs, in particular, offer advantages such as solution processability, simple fabrication, and low fabrication costs [1]. Traditional infrared photodetectors based on materials like InGaAs and HgCdTe suffer from limitations, including high costs, complex fabrication processes, and a limited tunability of the bandgap [5,10]. Despite these advantages, the performance of photodetectors with only PbS QDs as the detection medium is insufficient to meet the requirements of high-performance photodetectors due to their relatively low carrier mobility, necessitating the integration of additional materials to enhance the electrical performance [2].

Recently, hybrid two-dimensional heterostructures have emerged as a promising class of materials for photoelectric detection. These systems encompass various hybrid configurations, including 2D/bulk [11], 2D/QD (0D) [12], and 2D/nanowire (1D) [13] structures. Notably, 2D/0D hybrid systems are particularly promising as the synergy between the distinct properties of the two materials can facilitate novel functionalities that are unattainable with a single material [14]. The combination of quantum dots and graphene, with their different work functions and carrier concentrations, facilitates photoinduced charge transfer, thereby enhancing the performance of photodetectors [15,16]. This combination compensates for the low carrier mobility of quantum dots and addresses the issues of weak light absorption, rapid carrier recombination, and an insufficient gain mechanism in graphene [2]. Although research on the optoelectronic response of PbS quantum dot/graphene heterojunctions is well developed [1,3], the underlying physical mechanism related to the electrical transport remains inadequately explored. A comprehensive and systematic investigation of the electrical transport properties of PbS QD/graphene heterostructures is crucial to understand the evolution of their conductivity and carrier mobility under various conditions. This knowledge can be utilized to adjust the device structure, thereby improving the optoelectronic conversion efficiency, response speed, and sensitivity, which are essential for the development of high-performance photodetectors and optoelectronic switches [17]. Additionally, a comprehensive understanding of the electrical transport properties helps to identify and address problems that affect the stability of the devices [18].

In this paper, the infrared optical and electrical transport properties of PbS QD/graphene heterostructures are investigated. The heterostructure was fabricated by spin-coating PbS quantum dots on top of p-type heavily doped monolayer graphene, which was synthesized on a SiO_2_/Si substrate using chemical vapor deposition (CVD). It was then characterized using a low-temperature electrical transport measurement system. The study investigates the differences in electrical transport properties between the QD/graphene heterostructure and bare graphene, the relationship between the electrical transport properties and temperature variations, and the electrical transport properties of the QD/graphene heterostructure under different illumination conditions.

## 2. Materials and Methods

**Figure 1:**
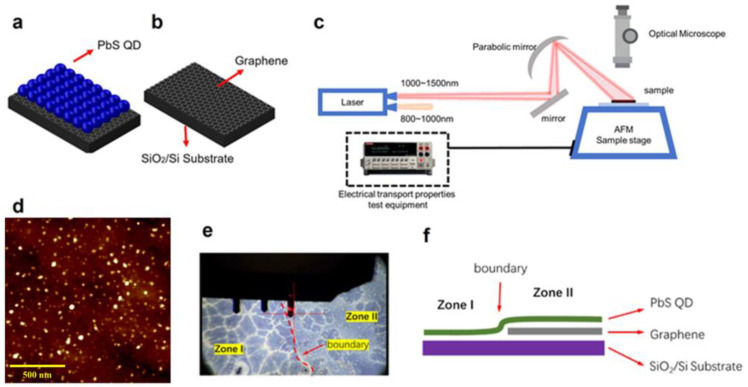
PbS QD/graphene heterojunction. (**a**) QD/graphene heterostructure device. (**b**) Bare graphene device. (**c**) Schematic diagram of the electrical transport measurement setup under infrared light excitation. (**d**) AFM image of PbS QD/graphene heterojunction. (**e**) Optical microscopy image of PbS QD/graphene heterojunction. (**f**) Schematic diagram of the horizontal structure for the QD/graphene heterojunction.

Figure 1a,b show schematic diagrams of the bare graphene and the QD/graphene heterostructure devices. The QD/graphene heterojunction device was fabricated in three steps. Initially, a p-type doped monolayer graphene film was deposited onto a SiO_2_/Si substrate using chemical vapor deposition (CVD). Subsequently, PbS quantum dots were spin-coated onto the graphene surface to form the QD/graphene/SiO_2_/Si heterostructure. Two distinct samples were prepared for this study, one before the spin-coating of QDs and the other after; these were designated as bare graphene and QD/graphene heterostructure devices, respectively. The PbS quantum dots were synthesized using the standard Schlenk line technique, with all reagents procured from Sigma Aldrich. This synthesis method is widely recognized for its robustness and reproducibility. According to characterization data from previous studies utilizing the same procedure, the average quantum dot diameter was estimated to be approximately 4 nm [19]. More detailed information on the fabrication and processing of the samples is provided in the Supplementary Materials.

**Figure 2:**
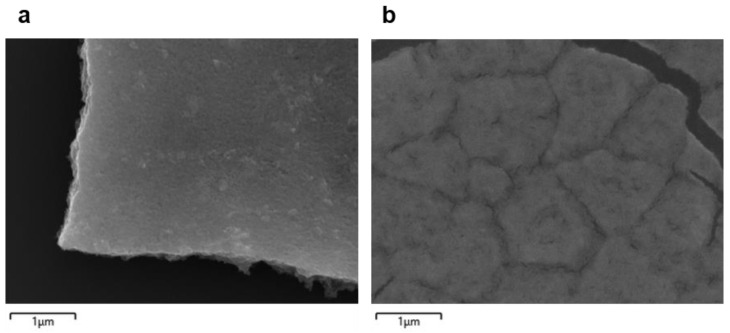
Scanning electron microscopy (SEM) images of the samples. (**a**) Graphene. (**b**) PbS QD/graphene heterostructures. Scale bar: 1 μm.

To evaluate the surface uniformity and flatness of graphene as well as the distribution uniformity of PbS quantum dots in the QD/graphene heterostructures, scanning electron microscopy (SEM) was used. As shown in Figure 2a, The SEM images of monolayer graphene, synthesized via CVD, revealed a continuous, defect-free structure, indicating the high quality of the graphene. The elemental analysis confirmed the expected composition, with carbon constituting approximately 50.49 atomic percent, a signature for pristine graphene. For the PbS QD/graphene heterostructure, the SEM analysis demonstrated a uniform distribution of the PbS QDs on the graphene surface with strong adhesion between the layers, as shown in Figure 2b. The elemental composition analysis identified significant concentrations of lead (27.33 wt%) and sulfur (4.66 wt%), consistent with the formation of PbS QDs. These results confirmed the successful preparation of high-quality graphene and PbS QD/graphene heterostructures, making them suitable for the further exploration of their electronic and optical properties. Detailed elemental composition lists and energy dispersive X-ray spectroscopy (EDS) layered images are provided in the Supplementary Materials.

The electrical transport properties of the devices were measured using a Montana Instruments Cryogenic Integrated Physical Property Measurement System (MICIPPMS, Montana Instruments Co., Bozeman, MT, USA). This system not only provides a low-temperature environment, but also integrates with external electrical transport measurement equipment, enabling the tunable temperature control of samples or devices. The key specifications of the system include a temperature control range for the sample stage from 3.2 K to 350 K without any load, temperature stability with peak fluctuations less than 15 mK, and vibration stability of the sample stage with peak vibrations less than 5 nm. These features ensure temperature stability and low vibration levels, which are critical for accurate data acquisition. External electrical transport measurements were performed using Stanford Research Systems lock-in amplifiers (models SR830 and SR850) (SRS, Sunnyvale, CA, USA), a Stanford Research Systems current amplifier (model SR570), and a Keithley 2400 Series source meter (Keithley, Cleveland, OH, USA) to investigate the electrical transport properties under different gate voltages at various temperatures as well as the temperature-dependent electrical transport properties of the material.

To perform electrical transport measurements under near-infrared illumination, we added an optical path on the MICIPPMS, as shown in Figure 1c. The laser system featured two emission windows, emitting light in the 800–1000 nm and 1000–1550 nm ranges. The sample, placed on an AFM sample stage, was subjected to light focused onto its surface by an off-axis parabolic mirror. An optical microscope capable of near-infrared observation was positioned above the sample to monitor both the sample and the optical focusing condition. The sample was connected to the electrical transport measurement equipment via a chip pin socket, which was converted to a BNC port for measuring purposes. Figure 1d shows an atomic force microscope (AFM) image of the PbS QD/graphene heterojunction, with a scale bar of 500 nm. Figure 1e shows the optical microscopy image of the PbS QD/graphene heterostructure. Figure 1f shows a schematic diagram of the horizontal structure for the QD/graphene heterojunction.

Before presenting the experimental results and analysis, we need to clarify the relationship between the size, density, and optical activity of PbS quantum dots (QDs) as well as the role of surface passivation. The size of PbS QDs directly influences their optical properties due to quantum confinement effects, where smaller QDs exhibit larger bandgaps, leading to blue shifts in the absorption and emission spectra. However, as the QD size decreases, the surface-to-volume ratio increases, resulting in a higher density of surface defects or trap states. These trap states act as non-radiative recombination centers, significantly reducing the device efficiency by capturing charge carriers and impeding radiative recombination. To mitigate this, surface passivation is critical. Graphene, as an effective passivation material, particularly for the PbS(111) plane, reduces the density of trap states and minimizes the recombination sites by passivating the surface dangling bonds [20]. This enhances charge carrier mobility and reduces non-radiative recombination, thus improving the overall optical and electronic properties of QDs. The incorporation of graphene into a heterostructure provides a highly conductive pathway for charge carriers, further optimizing the performance of QD-based optoelectronic devices.

In addition, we used 1,2-ethanedithiol (EDT) short-chain molecules for ligand exchange in our experiments to improve the electronic coupling between QDs by reducing the interparticle distance, promoting more efficient charge transport while maintaining effective surface passivation. This process ensured that trap states were minimized and non-radiative recombination losses were further reduced.

The density of QDs is another crucial factor. A high density increases light absorption, but may also lead to QD aggregation, creating regions with poor passivation and additional trap states, ultimately enhancing non-radiative recombination. Conversely, a low QD density could lead to suboptimal light absorption. Therefore, optimizing QD density is necessary to achieve a balance between effective light absorption and minimal trap-state formation.

## 3. Results

In this work, we systematically measured and analyzed the electrical transport properties of the graphene heterostructure at different temperatures as well as under different electrical modulation and optical illumination conditions using the established experimental setup.

### 3.1. Temperature-Dependent I–V Curves

**Figure 3:**
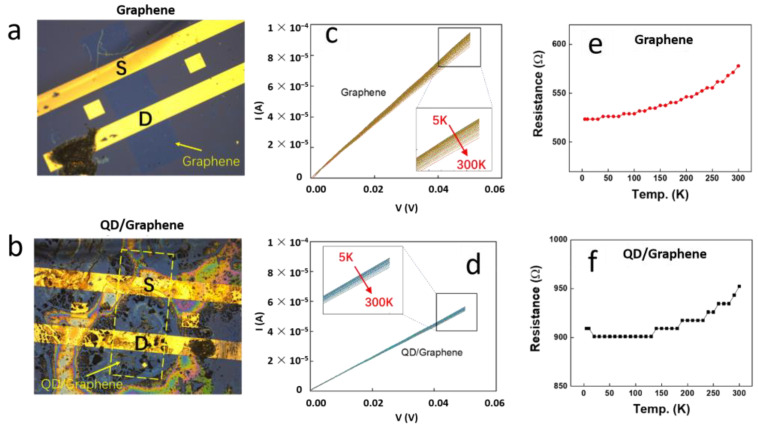
I–V curves of the graphene device and the QD/graphene heterostructure device at various temperatures. (**a**) Optical microscope image of the graphene device. The letter “S” represents the source electrode and the letter “D” represents the drain electrode. (**b**) Optical microscope image of the QD/graphene heterostructure device. (**c**) I–V curves of the graphene device at different temperatures. (**d**) I–V curves of the QD/graphene heterostructure device at different temperatures. (**e**) Temperature dependence of the resistance for the graphene device. (**f**) Temperature dependence of the resistance for the QD/graphene heterostructure device.

The dependence of the I–V curves for the graphene device and the QD/graphene heterostructure device on temperature was measured within a temperature range of 5 K to 300 K at intervals of 10 K. The results are presented in Figure 3.

Figure 3a,b illustrates the device configurations for the conducted measurements, with Figure 3a showing the graphene device and Figure 3b depicting the QD/graphene heterostructure device. As the graphene in the QD/graphene heterostructure was covered by QDs, the location of the graphene is marked with a yellow dashed box in Figure 3b, and the source (S) and drain (D) electrodes are also indicated. The configuration of the heterostructure device may have been an underlying factor affecting its electrical transport properties. The electrical transport results in Figure 3c,d shows that from 5 K to 300 K, the I–V curves of both devices were linear, indicating a good linear relationship within the tested temperature range. The data revealed that the resistance of the QD/graphene heterostructure was higher than that of graphene. The increased electrical resistance in the PbS/graphene heterostructure devices was primarily due to the interaction between the PbS QDs and graphene, which formed heterojunctions. Upon the deposition of PbS QDs, the p-doping in graphene is partially neutralized, shifting the Fermi level and Dirac point towards charge neutrality. This reduces the carrier concentration and increases the resistance. Additionally, heterojunctions introduce potential barriers that disrupt charge transport by creating depletion and accumulation regions, further contributing to the resistance rise. The resistance of both devices increased slightly with temperature, likely due to an increase in the thermally excited carrier concentration and enhanced phonon scattering. The resistance at each temperature was obtained using linear fitting. Figure 3e,f show the temperature-dependent resistance curves of the two devices. As the temperature increased from 5 K to 300 K, the resistance of the graphene device increased from 525 Ω to 575 Ω while the resistance of the QD/graphene heterojunction device increased from 900 Ω to 950 Ω. These measurements provided the fundamental electrical parameters and performance characteristics of the devices. The higher resistance in the QD/graphene heterostructure was primarily caused by changes in the charge carrier concentration and the heterojunction effects, which together hindered efficient charge transport.

### 3.2. Temperature-Dependent Transport Properties

**Figure 4:**
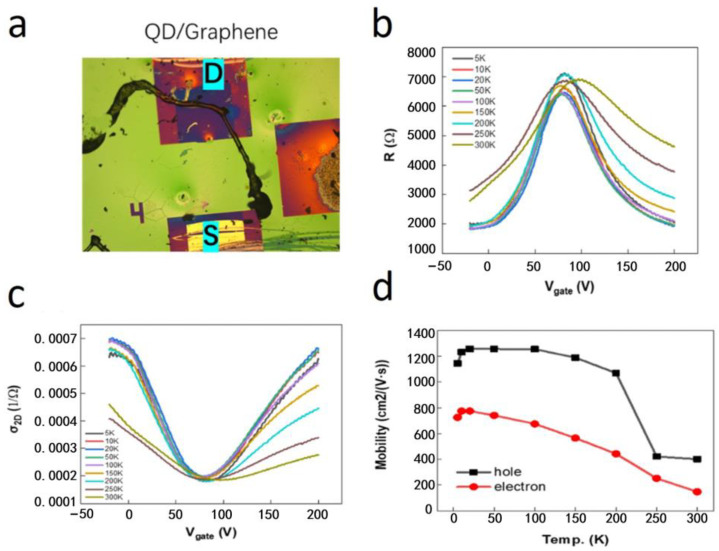
Electrical transport characteristics of the QD/graphene heterostructure device at various temperatures. (**a**) Optical microscope image of the device. The letter “S” represents the source electrode and the letter “D” represents the drain electrode. (**b**) Dependence of the resistance on the gate voltage at different temperatures. (**c**) Dependence of the two-dimensional conductivity on the gate voltage at different temperatures. (**d**) Temperature dependence of the carrier mobility.

The relationship between the resistance, two-dimensional conductivity, and gate voltage of the QD/graphene heterostructure devices was measured at different temperatures, with the temperature adjusted from 5 K to 300 K at intervals of 10 K. The results are presented in Figure 4.

Figure 4a displays the configuration of the measured device, with the source (S) and drain (D) electrodes indicated. Figure 4b,c show the relationship between the resistance and gate voltage as well as the dependence of two-dimensional conductivity on gate voltage, respectively, for the QD/graphene heterostructure device at various temperatures. The gate voltage *V_g_* can modulate the carrier concentration in graphene, and there is a linear relationship between the carrier concentration and the gate voltage, which is expressed as follows:

(1) $$dn=\alpha dV_{g},\alpha =\varepsilon /\left(d\cdot e\right)$$

Here, *n* represents the carrier concentration, *ε* is the dielectric constant, *d* is the thickness of the dielectric layer (with a SiO_2_ layer thickness of 500 nm), and *e* is the elementary charge. Upon the calculation, the value of *α* is approximately 4.1 × 10^10^ cm^−2^V^−1^. The carrier mobility can then be estimated using the following expression from the standard transistor model:

(2) $$\mu =d\sigma /dn\cdot 1/e=d\sigma /dV_{g}\cdot 1/\left(\alpha e\right)$$

In the expression, *σ* represents the conductivity between the channels of the field-effect transistor device. Figure 4b illustrates that the gate voltage could regulate the resistance within the QD/graphene heterostructure. Due to the p-type doping of graphene during device fabrication, the Fermi level of graphene is lowered and the hole concentration is higher than the electron concentration, with holes being the dominant charge carriers for conduction. As the gate voltage increases, the Fermi level rises, leading to a decrease in the hole concentration and, consequently, an increase in the resistance of the QD/graphene heterostructure. A further increment of the gate voltage causes the Fermi level to continuously rise. Therefore, the electron concentration in the device increases. The device then transits to the electron conduction domain and the resistance starts to decrease [21,22]. The experimental results from our study on a PbS QD/graphene heterostructure revealed that the gate voltage dependence was broadly consistent with the known properties of bare graphene. Previous studies have explored the relationship between the resistance and gate voltage in bare graphene at different temperatures [23,24]. Specifically, as the temperature increases, the resistance peak becomes asymmetric and has a decreased height and a broadened width. In the PbS QD/graphene heterostructures investigated in this study, we observed similar overall trends in the resistance versus gate voltage curves, indicating that the basic transport mechanism remained unchanged. However, the interaction between the PbS QDs and graphene introduced notable differences. Most importantly, a resistance peak appeared at gate voltages of tens of volts due to the p-type doping of graphene and maintained its height at elevated temperatures. This indicated that the heterostructure exhibited enhanced thermal stability at the charge neutrality point (Dirac point) compared with the bare graphene. In addition, the peak shifted to higher gate voltages as the temperature increased, a behavior we attributed to the interaction between the PbS QDs and the graphene layer, which affected the p-type doping level and the charge carrier distribution. Upon the integration of PbS QDs with graphene to form a heterostructure, well-aligned band edges between the PbS QDs and graphene facilitate the transfer of holes from PbS to graphene, thereby increasing the hole concentration and the conductivity of graphene [25]. With an increase in temperature, thermal excitation enhances the probability of holes overcoming the potential barrier at the interface between PbS QDs and graphene, leading to an increased influx of holes into the graphene. This accounts for the shift of the charge neutrality point to higher gate voltages as temperature increases, as depicted in Figure 4b–d, which demonstrate the temperature dependence of the carrier (holes and electrons) mobility in the QD/graphene heterostructure sample. It was observed that the electron mobility was lower than the hole mobility, and both carrier mobilities decreased with an increase in temperature. When the temperature increased from 5 K to 300 K, the hole mobility decreased from 1200 cm^2^V^−1^s^−1^ to 400 cm^2^V^−1^s^−1^ and the electron mobility decreased from 800 cm^2^V^−1^s^−1^ to 200 cm^2^V^−1^s^−1^. This temperature-dependent reduction in carrier mobility could be attributed to increased electron–phonon scattering at higher temperatures, which reduced the carrier mobility.

To summarize, although the general rules of the gate voltage modulation of the PbS QD/graphene heterostructure were consistent with that of bare graphene, the distinct differences in the magnitude and thermal stability as well as the shift in the Dirac point highlighted the significant impact of the PbS quantum dots on the overall transport characteristics. These findings provided a clearer understanding of how the quantum dot/graphene interactions influenced the electrical properties of the heterostructures, distinguishing them from the bare graphene.

### 3.3. Influence of Near-Infrared Illumination on Device Resistance

**Figure 5:**
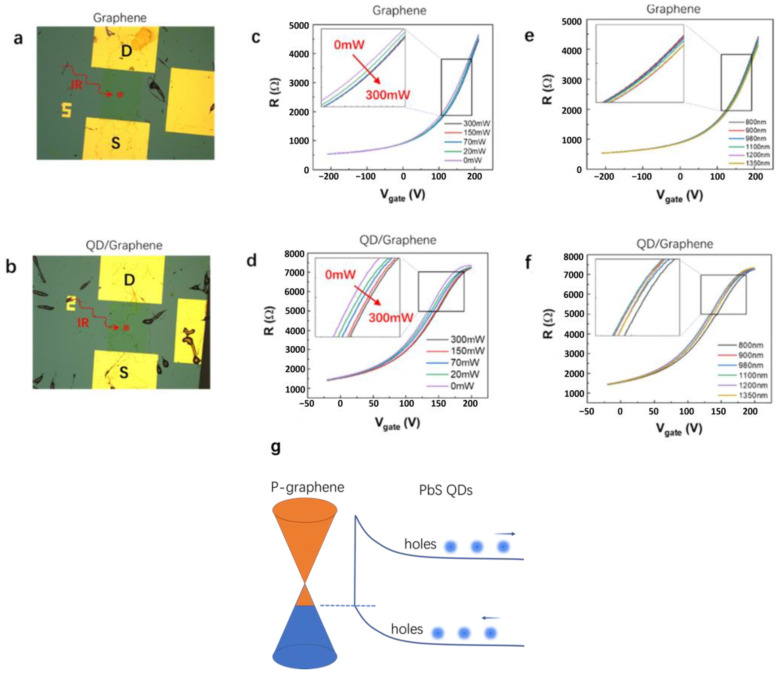
Electrical transport characteristics of graphene and QD/graphene heterostructure samples under different near-infrared illumination conditions. (**a**,**b**) Optical microscope images of the corresponding devices, with the light focus position (indicated by red dots), source (S), and drain (D) electrodes marked. (**c**,**d**) Resistances versus gate voltage curves at different illumination powers with a fixed illumination wavelength of 980 nm. (**e**,**f**) Resistances versus gate voltage curves at different illumination wavelengths at an illumination power of approximately 50 mW. (**g**) The schematic diagram of the interaction mechanism between PbS quantum dots and graphene. The orange area represents the graphene conduction band, and the blue area represents the graphene valence band.

The influence of near-infrared illumination on the transport properties was investigated using two groups of experiments where the transport properties of the graphene and QD/graphene heterostructures were compared. In the first group of experiments, the relationship between the resistance and the gate voltage was measured at different illumination powers (0 mW, 20 mW, 75 mW, 150 mW, and 300 mW) when the illumination wavelength was fixed at 980 nm. In the second group of experiments, the illumination power was instead fixed at 50 mW and the resistances at different gate voltages were measured while the illumination wavelength was set at 800 nm, 900 nm, 980 nm, 1100 nm, 1200 nm, and 1350 nm, respectively. The results are presented in Figure 5.

Figure 5a represents the graphene device, while Figure 5b depicts the QD/graphene heterostructure device. The source (S) and drain (D) electrodes are marked, and the position of the light focus is indicated by red dots. With a fixed illumination wavelength of 980 nm, resistance–gate voltage curves were obtained for the two devices at different illumination powers. Due to the p-type heavy doping of graphene, a gate voltage higher than 200 V was required to observe the resistance extremum. Additionally, the resistance of the QD/graphene heterostructure was higher than that of bare graphene. Figure 5c,d illustrate the relationship between the illumination condition and the resistance of the two samples. Near-infrared illumination had a relatively smaller effect on the resistance of the graphene device compared with the QD/graphene heterostructure device. As shown in Figure 5d, under the same gate voltage, an increase in the illumination power led to a decrease in the resistance of the QD/graphene heterostructure device. This was likely due to the generation of more carriers by illumination, which increased the conductivity and reduced the resistance. At the steepest part of the curve (where the slope was at the maximum and the gate voltage was approximately 125 V), the resistance variation of the device was approximately 0.4%/mW. Under the condition of a fixed illumination power of 50 mW, the change in the illumination wavelength had a smaller effect on the resistance of the graphene device, as shown in Figure 5e, while it had a relatively larger effect on the resistance of the QD/graphene heterostructure device, as shown in Figure 5f. These phenomena indicated that the quantum dots in the QD/graphene heterostructure device enhanced the absorption of incident light in this wavelength range.

The schematic diagram of the interaction mechanism between PbS quantum dots and graphene is shown in Figure 5g. PbS QDs display quantum confinement due to their nanoscale sizes, yielding discrete energy levels [26]. The integration of PbS QDs with graphene results in a work function mismatch, creating an internal electric field that induces band-bending at the interface, thereby achieving optimal band alignment [22,25,27]. Under illumination, PbS QDs generate electron-hole pairs that are effectively separated at the interface, with the aligned bands aiding the transfer of photogenerated holes to graphene, augmenting its hole density [22,25,26,27]. This transfer is accompanied by a reduction in the graphene’s Fermi level, thereby enhancing the electrical conductivity of a device.

The optoelectronic properties of QDs are influenced by the generation of electron-hole pairs through photon absorption. Increasing the light intensity or frequency can stimulate the generation of more electron-hole pairs within the QD. This is consistent with the results presented in Figure 5d,f [28]. Furthermore, the charge transfer between PbS QDs and graphene induces a photogating effect; that is, the resistance of a QD/graphene heterostructure is modulated by the illumination conditions. The resistance variation range can be fine-tuned by adjusting the gate voltage. These observations suggest the potential of PbS QD/graphene heterostructures for photodetection applications [22].

The measurement results demonstrated that the electrical transport characteristics of the QD/graphene heterostructure exhibited significant sensitivity to near-infrared illumination conditions. The resistance of the QD/graphene heterostructure varied with the illumination power and wavelength, highlighting its sensitivity to illumination conditions. Furthermore, the influence of illumination on the resistance of the QD/graphene heterostructure device was greater than that on the graphene device, which could be attributed to an increased absorption and a higher conversion efficiency of light by the quantum dots in the QD/graphene heterostructure. These findings not only revealed the electrical transport characteristics of the QD/graphene heterostructure under near-infrared illumination conditions, but also provide important experimental evidence for the design and optimization of infrared photodetectors based on QD/graphene heterostructures.

## 4. Conclusions

In this paper, PbS QD/graphene heterostructures were fabricated and their electrical transport properties were comprehensively investigated. A key focus was temperature-dependent electrical transport in the PbS QD/graphene heterostructures, highlighting the crucial impact of quantum dots on carrier mobility and resistance. The results offer novel insights for optimizing device stability under varying thermal conditions. Specifically, the resistance of the QD/graphene samples was higher than that of bare graphene, with both slightly increasing with temperature. When the temperature increased from 5 K to 300 K, the resistance of the graphene device increased from 525 Ω to 575 Ω, while the resistance of the QD/graphene heterojunction device increased from 900 Ω to 950 Ω. Notably, the QD/graphene samples exhibited pronounced hole doping, where the conductivity rose from 0.0002 Ω^−1^ to 0.0007 Ω^−1^ under a gate voltage modulation. With an increase in temperature from 5 K to 300 K, the hole mobility decreased from 1200 cm^2^V^−1^s^−1^ to 400 cm^2^V^−1^s^−1^ and the electron mobility dropped from 800 cm^2^V^−1^s^−1^ to 200 cm^2^V^−1^s^−1^, likely due to enhanced electron–phonon scattering. Furthermore, the tunability of the electronic properties for PbS QD/graphene heterostructures under different near-infrared illumination intensities and wavelengths was demonstrated, revealing their potential as tunable high-performance infrared photodetectors. Under near-infrared illumination, both the bare graphene and the QD/graphene heterostructure exhibited reduced resistances, indicating increased carrier generation and enhanced conductivity. At a gate voltage of approximately 125 V, the change in the resistance of the QD/graphene heterostructure device due to illumination was about 0.4%/mW, indicating its great potential in infrared photodetector applications.

## Data Availability

The data presented in this study are available on request from the corresponding author.

## Supplementary Materials

The following supporting information can be downloaded at: https://www.mdpi.com/article/10.3390/nano14201656/s1, Figure S1: Image of the sample electrode under optical microscopy. (a) after development and fixing. (b) after evaporation deposition. Figure S2: SEM characterization of bare graphene. (a) EDS layered image. (b) electronic image. (c–e) Corresponding elemental mappings image of Si, O and C. (f) Spectrogram of the total number of distribution maps. Figure S3: SEM characterization of PbS QD/graphene heterostructure. (a) EDS layered image. (b) electronic image. (c–e) Corresponding elemental mappings image of C, Pb and S. (f) Spectrogram of the total number of distribution maps. Table S1: Spectrogram of the total number of distribution maps (bare graphene). Table S2: Spectrogram of the total number of distribution maps (PbS QD/graphene heterostructure).
