# Supplementary material for: Electrical Transport Properties of PbS Quantum Dot/Graphene Heterostructures
Source: Nanomaterials (Basel). 2024 Oct 16;14(20):1656. doi: 10.3390/nano14201656 (PMC11510120; doi:10.3390/nano14201656)
Supplement: Supplementary file 1 [file nanomaterials-14-01656-s001.zip › nanomaterials-3195314-supplementary.pdf]

Supplementary Information

Electrical Transport Properties of PbS Quantum Dot/Graphene Heterostructures

**Haosong Ying [1], Binbin Wei [2], Qing Zang [2,*], Jiduo Dong [1], Hao Zhang [1], Hao Tian [1], Chunheng Liu [2]
and Yang Liu [2,*]**

[1] Department of Physics, Harbin Institute of Technology, Harbin 150001, China;
24b911011@stu.hit.edu.cn (H.Y.); 19b911015@stu.hit.edu.cn (J.D.); 19b911016@stu.hit.edu.cn (H.Z.); tianhao@hit.edu.cn (H.T.)

[2] Institute of System Engineering, Academy of Military Sciences, Beijing 100191, China; weibb.2009@tsinghua.org.cn (B.W.); liuchunheng@126.com (C.L.)

* Correspondence: 1101100207@alu.hit.edu.cn (Q.Z.); lyang.2003@tsinghua.org.cn (Y.L.)

1. Synthesis of PbS quantum dots

In this work, PbS quantum dots (QDs) are synthesized using the hot injection method. Initially, 2 mmol of lead oxide (PbO) and 4.7 mmol of oleic acid (OA) are dissolved in 20 mL of 1-octadecene (ODE), which serves as both the solvent and reaction medium. The mixture is degassed under vacuum at 95°C for 12 hours. Following this, the temperature is increased to 120°C, and 0.01 mmol hexamethyldisilathiane (HMS) mixed with 10 ml of ODE is rapidly injected to trigger the formation of PbS quantum dots. The system is then cooled gradually under the control of the heating mantle, taking approximately 1 hour to reach room temperature. Afterward, acetone and ethanol (5:1) are added, and the QDs are isolated by centrifugation. The QDs are further purified through two rounds of dispersion and precipitation and ultimately dispersed in anhydrous n-octane.

2. Fabrication of the electrical transport test samples

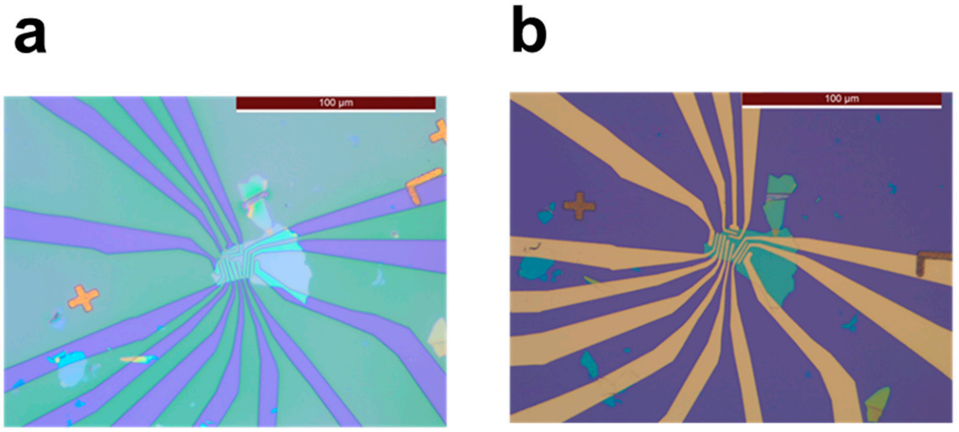

Figure S1 Image of the sample electrode under optical microscopy. (a) after development and fixing. (b) after evaporation deposition

Fabrication of the electrical transport test samples involves a meticulous process to prepare field-effect transistor (FET) devices, utilizing SiO_2/Si substrates layered with two-dimensional materials such as graphene and graphene heterostructures. Initially, the samples are cut and spin-coated with a 400 nm thick layer of PMMA (PMMA-A6), followed by a baking process at 180°C for two minutes. Subsequently, the sample surface is marked under a 2X optical microscope using a probe station, with the PMMA being scratched in an area 500 μm away from the target sample. Electron beam lithography (EBL) is then employed to create local marks around the target area using a GeminiSEM300 system (Zeiss) with a 300 μm write field and a 15 pA beam current. After development and fixing, a chromium-gold alloy is evaporated onto the marked areas and lifted off using a mixture of acetone and isopropanol. The optical microscopy image of the graphene sample after development exposure is shown in Figure S1a, and the optical microscopy image of the electrode after evaporation deposition is presented in Figure S1b. This sequence is repeated, with EBL used to define the graphene edges for etching, ensuring the geometric integrity of the transistor channel. Oxygen plasma etching at 40 W and 5 sccm is applied afterward to clean the graphene edges, with the cleanliness confirmed by SEM imaging.

For the lead wire processing, the samples with gold electrodes are mounted onto chip carriers and connected to external circuits using a wire bonding technique. Aluminum wires are utilized for their reliable electrical conductivity and ease of bonding. The wire bonding is carried out with a wire bonder system that employs

ultrasonic energy to securely attach the aluminum wires to the gold electrodes, ensuring strong electrical connections while minimizing the risk of damaging the SiO_2 dielectric layer. To further protect the integrity of the connections, conductive silver paste is applied at the bonding sites, providing both mechanical stability and enhanced conductivity. This careful preparation of the FET devices is essential for accurate and reliable electrical transport measurements in subsequent experimental analyses.

Scanning electron microscopy (SEM) image of the samples

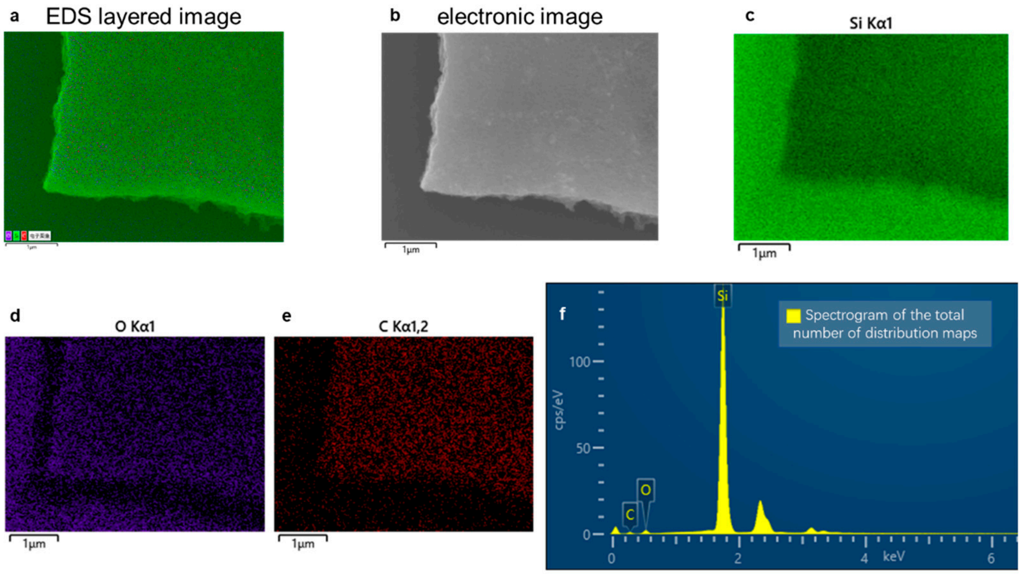

Figure S2 SEM characterization of bare graphene. (a) EDS layered image. (b) electronic image. (c-e) Corresponding elemental mappings image of Si, O and C. (f) Spectrogram of the total number of distribution maps.

Table S1 Spectrogram of the total number of distribution maps (bare graphene)

Element	Line Type	Apparent concentration	k-ratio	Wt%	Wt% Sigma	At%
C	K-line	0.81	0.00813	32.92	0.65	50.49
O	K-line	1.83	0.00615	11.13	0.19	12.81
Si	K-line	35.58	0.28195	55.95	0.55	36.70
Total				100.00		100.00

Figure S2 shows SEM images of CVD graphene. As shown in Figure S2a, the EDS layered image of bare graphene demonstrates the existence of the Si, O and C elements. The element distribution image clearly delineates the boundary between graphene and the SiO_2 substrate. The surface morphology of CVD graphene is also observed in Figure S2b. In the SEM image, graphene appears as a light, continuous film with a smooth

surface and a relatively uniform thickness. The boundary between graphene and SiO_2 substrate is visible. Figure S2c-e presents the element mapping images of corresponding Si, O and C, illustrating their distribution. The uniform distribution of carbon also reveals a uniform graphene thickness. The EDS results of the bare graphene indicate the presence of Si, O and C in the graphene device (Figure S2f). The elemental analysis confirms the expected composition, with carbon constituting approximately 50.49 atomic percent, a signature for pristine graphene (as shown in Table S1).

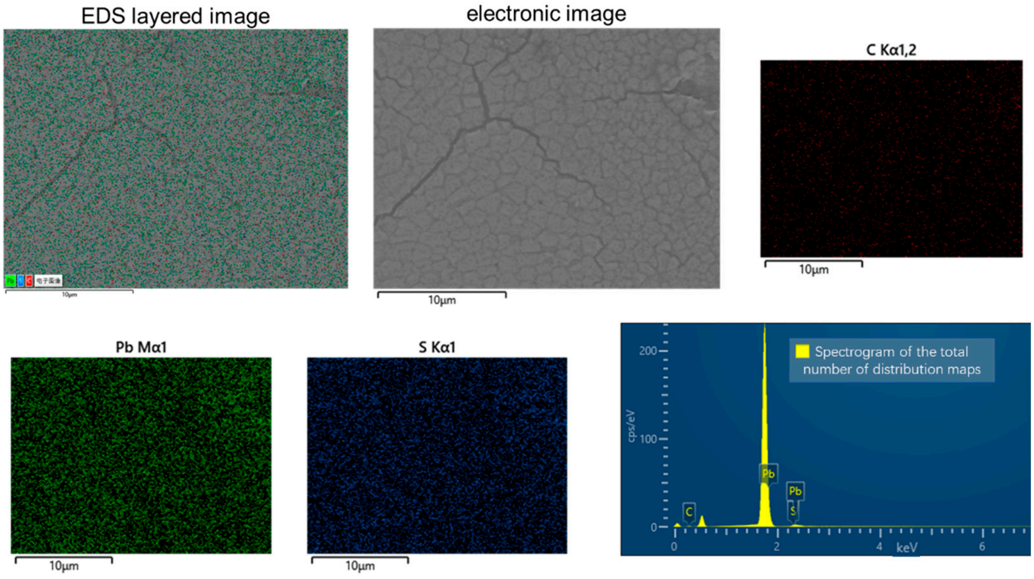

Figure S3 SEM characterization of PbS QD/graphene heterostructure. (a) EDS layered image. (b) electronic image. (c-e) Corresponding elemental mappings image of C, Pb and S. (f) Spectrogram of the total number of distribution maps.

Table S2 Spectrogram of the total number of distribution maps (PbS QD/graphene heterostructure)

Element	Line Type	Apparent concentration	k-ratio	Wt%	Wt% Sigma	At%
C	K-line	0.45	0.00453	68.01	1.29	95.33
S	K-line	0.17	0.00149	4.66	0.31	2.45
Pb	M-line	0.95	0.00887	27.33	1.21	2.22
Total				100.00		100.00

Figure S3 shows SEM images of the PbS QD/graphene heterostructure. As shown in Figure S3a, the EDS layered image of QD/graphene heterostructure demonstrates the uniform distribution of the C, Pb and S elements. The surface morphology of PbS QD/graphene heterostructure is observed in Figure S3b. Figure S3c-e presents the element mapping images of C, Pb and S, describing the distribution of C, Pb and S. The uniform distribution of Pb and S indicates that the PbS QDs are uniformly spin-coated

on graphene. The EDS results of the PbS QD/graphene heterostructure indicate the presence of C, Pb and S in the QD/graphene heterostructure device (Figure S3f). The elemental composition analysis identifies significant concentrations of lead (27.33 weight percent (wt%)) and sulfur (4.66 wt%), confirming the formation of PbS QDs (as shown in Table S2).
